# Molecular Evidence of Drug Resistance in Asymptomatic Malaria Infections, Myanmar, 2015 

**DOI:** 10.3201/eid2303.161363

**Published:** 2017-03

**Authors:** Myat Htut Nyunt, Thinzar Shein, Ni Ni Zaw, Soe Soe Han, Fauzi Muh, Seong-Kyun Lee, Jin-Hee Han, Kyaw Zin Thant, Eun-Taek Han, Myat Phone Kyaw

**Affiliations:** Department of Medical Research, Yangon, Myanmar (M.H. Nyunt, T. Shein, N.N. Zaw, S.S. Han, K.Z. Thant, M.P. Kyaw);; Kangwon National University, Chuncheon, South Korea (M.H. Nyunt, S.-K. Lee, J.-H. Han, F. Muh, E.-T. Han)

**Keywords:** malaria, antimicrobial resistance, Myanmar, asymptomatic, artemisinin, plasmodium, parasites, PCR

## Abstract

Artemisinin resistance containment in Myanmar was initiated in 2011 after artemisinin-resistant *Plasmodium falciparum* malaria was reported. Molecular evidence suggests that asymptomatic malaria infections harboring drug resistance genes are present among residents of the Myanmar artemisinin resistance containment zone. This evidence supports efforts to eliminate these hidden infections.

The global burden of malaria has been decreasing in recent years as a result of high levels of control of the spread of infection, and the ultimate goal of malaria elimination by 2030 in all Greater Mekong Subregion countries in Southeast Asia seems attainable (*1*). However, artemisinin-resistant *Plasmodium falciparum* malaria has been reported in Cambodia, Thailand, Myanmar, Laos, and Vietnam (*2*). Chloroquine-resistant *P. vivax* malaria has also been confirmed in 10 countries, including Myanmar (*3*), and mutations in the mefloquine-resistance molecular marker (*pvmdr1* mutation) and sulfadoxine/pyrimethamine-resistance markers (*pvdhps, pvdhfr* mutations) have been reported in Myanmar (*4*).

A containment program for artemisinin-resistant malaria was initiated in 2011 according to the Global Plan for Artemisinin Resistance Containment. Areas where artemisinin resistance was documented were ranked as Tier I under the protocol, whereas areas where resistance was suspected were ranked as Tier II. After Myanmar artemisinin resistance containment (MARC) was initiated, malaria morbidity and mortality rates decreased dramatically, especially in MARC Tier I areas (*5*). However, there are no reports on the prevalence of asymptomatic infections, which may represent a reservoir of local malaria transmission. In this study, we aimed to determine the prevalence of asymptomatic malaria infection and to analyze drug-resistance markers in asymptomatic *P. falciparum* and *P. vivax* infections.

## The Study

As of 2014, the Tier I area of artemisinin resistance in Myanmar was composed of 52 townships; the remaining regions were designated as Tier II. In January 2015, we conducted a cross-sectional study of one of the Tier I areas of the MARC, Shwegyin Township (22°20′0″N, 95°56′0″E) (Figure; Technical Appendix).

**Figure:**
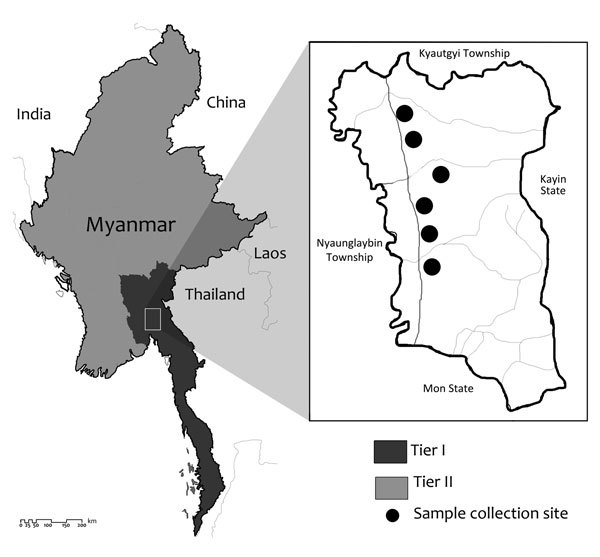
The study site, Shwegyin Township, Myanmar, where molecular evidence of drug resistance in asymptomatic malaria infections was obtained. As of 2014, Myanmar artemisinin resistance containment areas were divided into Tier I (52 townships) and Tier II (all remaining townships).

Rapid diagnostic tests (RDTs) (HRP2 and *P. vivax*–specific pLDH-based RDT, SDFK80; Standard Diagnostics, Gyeonggi-do, South Korea), microscopy, and PCR were used to screen for asymptomatic malaria infection (online Technical Appendix). We examined 1,182 local residents, with a male:female ratio of 4:5 and a median age of 30 years (interquartile range 18–45 years). Among these residents, 549 (46.4%) had a history of malaria infection within the past 5 years. No clinical cases of malaria infection were detected during the survey period. Although we found no RDT-positive cases of malaria infection, we detected 2 *P. vivax* infections by microscopy, with parasite densities of 580 and 1,200 parasites/µL.

When we performed molecular detection for the 4 common malaria species (online Technical Appendix), the overall rate of asymptomatic malaria infection was 2.4% (28/1,180) and included 4 *P. falciparum*, 22 *P. vivax*, and 2 *P. malariae* infections. Although the overall prevalence of asymptomatic infection in these areas was not high, it was similar to that observed in the Thailand–Myanmar border area during 2013–2014 (*6*).

In this study, RDT and microscopy missed almost all the asymptomatic infections detected by PCR, indicating that only the molecular method is suitable for the detection of asymptomatic infections. Moreover, the asymptomatic cases were broadly distributed geographically throughout the study area. Most of the infections were in male patients (19/28, 67.8%) and in the working-age group. Neither sex nor occupation was identified as an associated factor for asymptomatic infection (Technical Appendix Table 2).

The established artemisinin-resistance marker K13 (kelch 13 gene) and the associated markers *pfarps10* (*P. falciparum* apicoplast ribosomal protein S10), *pffd* (*P. falciparum* ferredoxin), and *pfmdr2* (*P. falciparum* multidrug-resistance protein 2) were analyzed in all asymptomatic *P. falciparum* cases. Nonsynonymous mutations in the propeller region of K13 were found to be associated with artemisinin resistance and associated delayed clearance of the parasite beyond 72 hours after treatment with artemisinin-based combination therapy (*7*). A previous study in the same region of patients with uncomplicated *P. falciparum* malaria indicated that 25.3% carried mutant K13 alleles (*8*). Markers that showed the underlying genetic background predisposing to the K13 mutant were also reported, including *pfarps10, pffd, pfmdr2*, and *pfcrt*. Specific single nucleotide polymorphisms of these genes, such as V127M of *pfarps10,* D193Y of *pffd*, and T484I of *pfmdr2*, were found at a similar prevalence as K13 mutations (*9*).

Among the 4 asymptomatic *P. falciparum* infections, 2 isolates showed K13 mutations (C580Y in 1 isolate and P574L in the other). C580Y is a well-known validated mutation, and P574L is a candidate marker for artemisinin resistance. Both mutations were reported only in locations in Southeast Asia where artemisinin resistance has been identified (*2*). Moreover, we observed the *pfarps10* mutation (V127M) in 2 of the cases, the *pffd* mutation (D195Y) in 3 cases, and the *pfmdr2* (T484I) mutation in all 4 isolates (Table; Technical Appendix Table 3). This molecular evidence suggests the presence of artemisinin resistance in asymptomatic isolates and calls for action toward eliminating this parasite reservoir.

**Table:** *Plasmodium falciparum* and *P. vivax* drug-resistance molecular markers in asymptomatic infections, Myanmar, 2015

Target	Description*	No. isolates/total (%)
kelch 13 (K13)	Wild	2/4 (50.0)
	C580**Y**	1/4 (25.0)
	P574**L**	1/4 (25.0)
*P. falciparum* apicoplast ribosomal protein S10 (*pfarps10*)	Wild	2/4 (50.0)
	V127**M**	2/4 (50.0)
*P. falciparum* ferredoxin (*pffd*)	Wild	1/4 (25.0)
	D193**Y**	3/4 (75.0)
*P. falciparum* multidrug-resistance protein 2 (*pfmdr2*)	Wild	0/4 (0.0)
	T484**I**	4/4 (100.0)
*P. vivax* chloroquine-resistance transporter (*pvcrt-o*)	Wild	7/21 (33.3)
	Mutant (AAG insert)	14/21 (66.7)
*P. vivax* multidrug-resistance protein 1 (*pvmdr1*)	Wild (T, Y, F) (958, 976, 1076)	0/21 (0.0)
	Double mutant (**M**,Y, **L**)	4/21 (19.0)
	Single mutant (**M**, Y, F)	12/21 (57.1)
	Triple mutant (**M**, **F**, **L**)	5/21 (23.8)
*P. vivax* dihydropteroate synthase (*pvdhps*)	Wild (S, A, K, A) (382, 383, 512, 553)	0/20 (0.0)
	Single mutant (S, **G**, K, A)	4/20 (20.0)
	Double mutant (S, **G**, K, **G**)	9/20 (45.0)
	Triple mutant (**A**, **G**, K, **G**)	5/20 (25.0)
	Quadruple mutant (**A**, **G**, **M**, **G**)	2/20 (10.0)
*P. vivax* dihydrofolate reductase (*pvdhfr*)	Wild (F, S, T, S) (57, 58, 61, 117)	0/21 (0.0)
	Single mutant (**L**, S, T, S)	1/21 (4.8)
	Double mutant (F, **R**, T, **N**)	2/21 (9.5)
	Quadruple mutant (**L/I**, **R**, **M**, **T**)	18/21 (85.7)

*Numbers in parentheses indicate the amino acid position. Mutant amino acids are shown in bold. All sequences were aligned with 3D7 (*P. falciparum*) and Sal-1 (*P. vivax*) reference sequences from http://www.plasmodb.org.

Similarly, we analyzed all available drug-resistance molecular markers in *P. vivax* (*10*), such as *pvcrt* (*P. vivax* chloroquine-resistance transporter), *pvdhps* (*P. vivax* dihydropteroate synthase), *pvdhfr* (*P. vivax* dihydrofolate reductase), and *pvmdr1* (*P. vivax* multidrug-resistance protein 1), in all *P. vivax* infections. We conducted analysis by using nested PCR, followed by gene sequencing (Technical Appendix).

Among the 22 asymptomatic *P. vivax* infections, we were unable to amplify *pvcrt-o, pvdhfr*, and *pvmdr1* in 1 isolate and *pvdhps* in 2 isolates. A high mutation rate was observed in known drug-resistance markers such as *pvcrt-o* K10 AAG insert (66.6%, 14/21), *pvdhps* (100.0%, 20/20), *pvdhfr* (100.0%, 21/21), and *pvmdr1* (100.0%, 21/21) (Table). Asymptomatic isolates in this study showed a higher mutation rate of the *pvcrt-o* AAG insert than those studied in neighboring countries such as Thailand (*11*), India (*12*), and China (*10*). In the *pvmdr1* gene, both Y976F and F1076L mutations were observed in 23.8% of cases and F1076L in 19.0% of cases; these rates were higher than those for China (*10*) and India (*12*) but lower than those for Thailand (*13*,*14*). Although antifolates are not the recommended antimalarial drugs for treatment of *P. vivax*, *pvdhfr* and *pvdhps* mutation rates were noticeable. This finding indicates that drug pressure in *P. vivax* malaria contributing to drug resistance also needs to be considered in addition to emphasizing the artemisinin-resistant *P. falciparum* malaria.

One limitation of this study is the exclusive focus on the local residents in the MARC area, where all available control and prevention measures had already been implemented. Unlike the mobile and migrant population, local residents have not been a top priority for the artemisinin resistance containment program, leading to a niche of hidden infection. Moreover, blood pooling before DNA extraction was used in this study for molecular detection of malaria infection. Although this method is not ultrasensitive, it has a higher sensitivity than RDT and microscopy. The hidden asymptomatic infections and associated molecular markers for drug resistance among the asymptomatic cases detected in this study represent a threat to containment and elimination efforts with regard to drug-resistant parasites.

## Conclusions

All countries in the Greater Mekong Subregion have set an ultimate goal of eliminating malaria by 2030. One of the main challenges to achieving this goal is hidden asymptomatic infection, which maintains a reservoir for local transmission of malaria (*15*). Critically, these asymptomatic infections may carry drug-resistance genes, including genes for artemisinin resistance. Our results indicated that drug-resistant malaria parasites may be spreading, even in the containment areas or (pre-)elimination areas; this issue should, therefore, be addressed at a policy level. Detection and elimination of asymptomatic infections are of vital importance. Our evidence highlights the need for a strategy for eliminating drug-resistant malaria in asymptomatic infections in the containment areas.

Technical Appendix Description of the study population and procedures, sampling and laboratory procedures, molecular markers for Plasmodium falciparum infection and P. vivax isolates, and sequence analysis and statistics, Myanmar, 2015.
