# Supplementary material for: Molecular Evidence of Drug Resistance in Asymptomatic Malaria Infections, Myanmar, 2015
Source: Emerg Infect Dis. 2017 Mar;23(3):517–20. doi: 10.3201/eid2303.161363 (PMC5382746; doi:10.3201/eid2303.161363)
Supplement: Technical Appendix — Description of the study population and procedures, sampling and laboratory procedures, molecular markers for Plasmodium falciparum infection and P. vivax isolates, and sequence analysis and statistics, Myanmar, 2015. [file 16-1363-Techapp-s1.pdf]

Article DOI: http://dx.doi.org/10.3201/eid2303.161363

Molecular Evidence of Drug Resistance in Asymptomatic Malaria Infections, Myanmar, 2015

Technical Appendix

Study Population

We conducted this study in Shwegyin Township, Bago Region (22°20′0″N, 95°56′0″E) in January 2015 (Figure 1). According to 2014 census data, 107,462 persons were living in an area of 2,440.1 km^2. Because of the nearby gold mine, many migrant workers were working in this area, and malaria was identified as one of the major diseases in Shwegyin (*1*). Since 2011, Shwegyin Township has been defined as a Tier I area of Myanmar artemisinin resistance containment because of the evidence of delayed parasite clearance in therapeutic efficacy studies conducted in Shwegyin during 2009–2010 (*2*,*3*) with molecular evidence of K13 mutations, an artemisinin-resistance marker (Nyunt et al., unpublished data). Shwegyin has been selected as one of the townships targeted for a malaria elimination program initiated in Myanmar. In 2001, 14.1% of outpatients and 20.1% of inpatients in Shwegyin Hospital were infected with malaria. The trend of prevalence has been decreasing, however, resulting in 11.3% of outpatients and 3.5% of inpatients infected with malaria in 2010 and 1.2% and 0.1%, respectively, infected with malaria in 2014.

Study Procedures

We recruited the participants in this study by randomized cluster sampling in Shwegyin Township. Inclusion criteria included a minimum age of 6 years and being local residents in the study area for more than 3 years; both sexes were included. Because we aimed to know the status of asymptomatic infections in local residents, the migrant or mobile population was excluded. Persons who currently showed signs and symptoms of malaria were also not included in this study.

We obtained written informed consent from all the participants. All the patients with parasite infections detected by any method (rapid diagnostic test [RDT], microscopy, or molecular method) were treated according to the National Malaria Treatment Guideline. This study was approved by the Ethical Committee of the Department of Medical Research, Myanmar (approval no. 49/Ethics-2014). The study was also registered with ClinicalTrial.gov (identifier NCT02708199).

Sample Size Determination

The prevalence of asymptomatic infection in the study population was unknown. Based on the previous studies (*4*,*5*) conducted in Southeast Asia, we assumed the maximum possibility of infection was 25% in the study site. A required sample size was calculated by anticipated population proportion of asymptomatic infection (25%) among the population of 107,462 (as of the 2014 census), with marginal error (2.5%), and 95% CI. The minimum sample size required was 1,141; in this study, 1,182 participants were involved.

Sampling Procedure

Randomized cluster sampling method was used in this study. Shwegyin Township was selected according to the rationale described previously. Two of the 4 local health centers were randomly selected. All villages belonging to the local health centers were listed and 6 villages were randomly selected. Among these villages, a sampling interval was calculated to get the required blood samples in the villages, which we determined to be at least 191 per village. Household visits or meeting places were used to collect the samples, depending on the convenience of the participants.

Laboratory Procedures

We collected 1 mL of venous blood from the participant’s forearm under aseptic conditions, using a disposable syringe. The blood was used for detection of asymptomatic infections.

Rapid Diagnostic Test

We detected malarial infection in all participants by *Pf*HRP2- and *Pv*-specific pLDH-based RDT (SDFK80; Standard Diagnostics, Gyeonggi-do, South Korea) and peripheral blood film examination to exclude malaria infection in the field. We used venous blood for malaria detection by RDT according to the manufacturer's instructions.

Malaria Microscopy

We followed the World Health Organization standardized protocol (*6*) for malaria microscopy. Briefly, 10% Giemsa was used to stain thick and thin blood films in the field for initial screening. In the main laboratory, another set of thick and thin blood films was stained with 3% Giemsa stain for confirmation and validation of the result. We prepared a fresh Giemsa stain dilution at least once a day and possibly more often, depending on the number of slides processed. We examined the Giemsa-stained thick and thin blood films at a magnification of 1,000× to identify the parasite species and to determine the parasite density. We calculated the parasite density, expressed as the number of asexual parasites per microliter of blood, by dividing the number of asexual parasites by the number of leukocytes counted and then multiplying by an assumed leukocyte density (6,000 leukocytes/µL). All slides were stained and checked by World Health organization–certified microscopists and validated by an expert microscopist. A blood slide was considered negative when examination of 1,000 leukocytes revealed no asexual parasites. Two qualified microscopists read all the slides independently, and parasite densities was calculated by averaging the 2 counts. Blood smears with discordant results (differences between the 2 microscopists' results in species diagnosis, in parasite density of >50%, or in the presence of parasites) were reexamined by a third, independent microscopist, and parasite density was calculated by averaging the 2 counts closest to each other.

Molecular Detection

We adapted the pooling strategy described previously (*7*). In brief, 20 µL from each of 10 samples was combined to make 1 pool of 200 µL whole blood. These pooled blood samples were designated for DNA extraction with QIAamp DNA Blood Mini Kit (QIAGEN, Hilden, Germany) and eluted into a final volume of 100 µL. Ten microliters of the DNA eluent was used for *Plasmodium* genus-specific amplification of the 18S rRNA gene, as described elsewhere (*8*). Only *Plasmodium*-positive pools were included to do individual DNA extraction again; 1 µL of

the DNA eluent was used for individual genus and species identification for asymptomatic malaria infection by using genus and specific primer pairs (*8*).

Artemisinin Resistance Molecular Markers for *P. falciparum* Infection

We analyzed all the samples that tested positive for *P. falciparum* malaria infection for artemisinin-resistance molecular markers (*9,10*) such as K13 kelch genes (PF3D7_1343700), *pfarps10* (PF3D7_1460900.1), *pffd* (PF3D7_1318100), and *pfmdr2* (PF3D7_1447900) by using the pairs of primers (Table 1. The details for amplification of these targets are available at our institutional web page (http://kmrl.kangwon.ac.kr/).

We performed amplification with an Accupower Premix (Bioneer, Daejon, South Korea) in a final volume of 20 µL. The final volume included 250 nmol/L of each primer, 0.25 mmol/L of each dNTP, 10 mmol/L Tris-HCl (pH 9.0), 30 mmol/L $MgCl_2$, 1.0 units of Taq polymerase, and 2 µL of genomic DNA template.

For nested-1 PCR amplification of the K13 kelch propeller gene, initial denaturation at 95°C for 5 min was followed by 35 cycles at 95°C for 30 s, 58°C for 1 min, 72°C for 1.5 min, and a final extension at 72°C for 10 min. Using 1 µL of the nested-1 PCR product as a template, we applied the same conditions for the nested-2 PCR except for an annealing temperature of 60°C for 1 min and 72°C for 1 min with 30 cycles.

For nested-1 PCR amplification of the *pfarps10* gene, initial denaturation at 95°C for 5 min was followed by 35 cycles at 95°C for 30 s, 58°C for 1 min, 72°C for 1.5 min, and a final extension of 72°C for 10 min. Using 1 µL of the nested-1 PCR product as a template, we applied the same conditions for the nested-2 PCR except for an annealing temperature of 62°C for 1 min and 72°C for 1 min with 30 cycles.

For nested-1 PCR amplification of the *pffd* gene, initial denaturation at 95°C for 5 min was followed by 35 cycles at 95°C for 30 s, 62°C for 1 min, 72°C for 1 min, and a final extension at 72°C for 10 min. Using 1 µL of the nested-1 PCR product as a template, we applied the same conditions for the nested-2 PCR except for an annealing temperature of 60°C for 1 min and 72°C for 1 min with 30 cycles.

For nested-1 PCR amplification of the *pfmdr2* gene amplification, initial denaturation at 95°C for 5 min was followed by 35 cycles at 95°C for 30 s, 58°C for 1 min, 72°C for 1.5 min, and a final extension at 72°C for 10 min. Using 1 μL of the nested-1 PCR product as a template, we applied the same conditions for the nested-2 PCR except for an annealing temperature of 60°C for 1 min and 72°C for 1 min with 35 cycles.

We visualized the PCR products by 1% agarose gel electrophoresis stained with 0.05% Redsafe dye (iNtRON Biotechnology, Daejeon, South Korea). We purified the PCR products by using a MEGA quick-spin Total Fragment DNA Purification Kit (iNtRON Biotechnology) and sequenced them with primers from a commercial sequencing company (Genotech, Daejeon, South Korea). We compared all the nucleotide and amino acid sequences with the reference sequence of 3D7 version 3 (www.plasmodb.org) and aligned them by using software in the Lasergene Genomic Suite (MegAlign, version 7.1; DNAStar, Madison, WI, USA).

Drug Resistance Molecular Marker Analysis for *P. vivax* Isolates

We further analyzed all the *P. vivax* isolates for *pvcrt-o* (*P. vivax* chloroquine resistance transporter gene, PVX_087980), *pvmdr1* (*P. vivax* multidrug resistance protein 1, PVX_080100), *pvdhps* (*P. vivax* hydroxymethyl pterinpyrophosphokinase dihydropteroate synthetase, PVX_123230), and *pvdhfr* (*P. vivax* dihydrofolate reductase thymidylate synthase, PVX_089950) by using the modified procedures described previously (*11*). We performed amplification by using an Accupower premix (Bioneer, Daejon, South Korea) in a final volume of 20 µL, which included 250 nmol/L of each primers, 0.25 mmol/L of each dNTP, 10 mmol/L Tris-HCl (pH 9.0), 30 mmol/L MgCl2, 1.0 units of Taq polymerase, and 2 µL of genomic DNA template and pairs of primers (Table 1).

For the *pvcrt-o* PCR amplification, initial denaturation at 94°C for 10 min was followed by 35 cycles at 94°C for 30 s, 60°C for 45 s, 72°C for 1 min, and a final extension at 72°C for 10 min for nested-1 PCR. Using 1 µL of the nested-1 PCR product as a template, we applied the same conditions for the nested-2 PCR except for an annealing temperature of 60°C for 45 s with 30 cycles of amplification.

For the *pvmdr1* PCR amplification, initial denaturation at 94°C for 10 min was followed by 35 cycles at 94°C for 30 s, 58°C for 45 s, 72°C for 2 min, and a final extension at 72°C for 10 min for nested-1 PCR. Using 1 µL of the nested-1 PCR product as a template, we applied the

same conditions for the nested-2 PCR except for an annealing temperature of 62°C for 45 s and extension at 72°C for 45 s with 33 cycles of amplification.

For the *pvdhps* PCR amplification, initial denaturation at 94°C for 10 min was followed by 40 cycles at 94°C for 30 s, 58°C for 45 s, 72°C for 1.5 min, and a final extension at 72°C for 10 min for nested-1 PCR. Using 1 μL of the nested-1 PCR product as a template, the same conditions were applied for the nested-2 PCR except for 35 cycles of amplification.

For the *pvdhfr* PCR amplification, initial denaturation at 94°C for 10 min was followed by 35 cycles at 94°C for 30 s, 58°C for 45 s, 72°C for 1 min, and a final extension at 72°C for 10 min for nested-1 PCR. Using 1 μL of the nested-1 PCR product as a template, we applied the same conditions for the nested-2 PCR except for an annealing temperature of 62°C for 30 s and extension at 72°C for 1 min with 30 cycles of amplification.

We visualized the PCR products by 1% agarose gel electrophoresis and staining with 0.05% Redsafe dye (iNtRON Biotechnology). We purified the PCR products by using a MEGA quick-spin Total Fragment DNA Purification Kit (iNtRON Biotechnology) and sequenced them with primers from a commercial sequencing company (Genotech).

Sequence Analysis and Statistics

We compared all the nucleotide and amino acid sequences with the reference sequence of K13 kelch protein (gene ID: PF3D7_1343700), *pfarps10* (PF3D7_1460900.1), *pffd* (PF3D7_1318100), *pfmdr2* (PF3D7_1447900), *pvcrt* (PVX_087980), *pvdhps* (PVX_123230), *pvdhfr* (PVX_089950), and *pvmdr1* (PVX_080100) from www.plasmodb.org and aligned them by using software in the Lasergene Genomic Suite (MegAlign, version 7.1). We used SPSS software (version 22.0, IBM SPSS Statistics, Armonk, NY, USA) for all statistical analysis. We compared the frequency of mutations and haplotypes of the target genes among the groups by using χ^2 and Fisher exact tests with a two-sided confidence interval at the 95% confidence level. The sequences were deposited at GenBank (accession nos. KX000945–KX000959 and KX384672–KX384687).

References

1. Township-Health-Department. Shwegyin Township Health Profile 2012: Department of Health, Myanmar; 2013.

2. World Health Organization. Global report on antimalarial drug efficacy and drug resistance: 2000–2010. Geneva, Switzerland; 2010 [cited 2016 Aug 16]. http://www.who.int/malaria/publications/atoz/9789241500470/en/

3. World Health Organization Country Office Myanmar. Strategic Framework for Artemisinin Resistance Containment in Myanmar (MARC) 2011–2015; 2011 [cited 2016 Aug 16]. http://www.searo.who.int/myanmar/documents/MARCframeworkApril2011.pdf

4. Imwong M, Nguyen TN, Tripura R, Peto TJ, Lee SJ, Lwin KM, et al. The epidemiology of subclinical malaria infections in South-East Asia: findings from cross-sectional surveys in Thailand–Myanmar border areas, Cambodia, and Vietnam. Malar J. 2015;14:381. http://dx.doi.org/10.1186/s12936-015-0906-x

5. Starzengruber P, Fuehrer HP, Ley B, Thriemer K, Swoboda P, Habler VE, et al. High prevalence of asymptomatic malaria in south-eastern Bangladesh. Malar J. 2014;13:16. http://dx.doi.org/10.1186/1475-2875-13-16

6. World Health Organization. Methods for surveillance of antimalarial drug efficacy. Geneva: WHO; 2009 [cited 2016 Aug 16]. http://www.who.int/malaria/publications/atoz/9789241597531/en/

7. Nyunt MH, Kyaw MP, Thant KZ, Shein T, Han SS, Zaw NN, et al. Effective high-throughput blood pooling strategy before DNA extraction for detection of malaria in low-transmission settings. Korean J Parasitol. 2016;54:253–9. http://dx.doi.org/10.3347/kjp.2016.54.3.253

8. Snounou G, Singh B. Nested PCR analysis of *Plasmodium* parasites. Methods Mol Med. 2002;72:189–203. http://dx.doi.org/10.1385/1-59259-271-6:189

9. Ariey F, Witkowski B, Amaratunga C, Beghain J, Langlois AC, Khim N, et al. A molecular marker of artemisinin-resistant *Plasmodium falciparum* malaria. Nature. 2014;505:50–5. http://dx.doi.org/10.1038/nature12876

10. Miotto O, Amato R, Ashley EA, MacInnis B, Almagro-Garcia J, Amaratunga C, et al. Genetic architecture of artemisinin-resistant *Plasmodium falciparum.* Nat Genet. 2015;47:226–34. http://dx.doi.org/10.1038/ng.3189

11. Lu F, Wang B, Cao J, Sattabongkot J, Zhou H, Zhu G, et al. Prevalence of drug resistance-associated gene mutations in *Plasmodium vivax* in central China. Korean J Parasitol. 2012;50:379–84. http://dx.doi.org/10.3347/kjp.2012.50.4.379

Technical Appendix Table 1. Pairs of primers used for amplification of the drug resistance markers for asymptomatic malaria, Myanmar, 2015

Target gene	PCR	Primer	Primer sequences, 5′→3′
K13 (PF3D7_1343700)	Nested 1	K13_1R	CGG AGT GAC CAA ATC TGG GA
		K13_3NF	AGC GGA AGT AGT AGC GAG AA
	Nested 2	K13_2F	GCC AAG CTG CCA TTC ATT TG
		K13_3R	GCC TTG TTG AAA GAA GCA GA
pfarps10 (PF3D7_1460900.1)	Nested 1	PfARPS_F3	TGC GAC TTT TAG GGT GTG GA
		PfARPS_N2R	CAT GGT ACC ACT TTT TCT TTT CCA
	Nested 2	PfARPS_F1	TTG TAG CAG GCC CAA TTC CC
		PfARPS_N2R2	TCT GGG TAA TTT GAC ATT CAT
pffd (PF3D7_1318100)	Nested 1	PfFD_F1	AGT TGT TCT ACA TGC GCA GC
		PfFD_R1	AAT GTG CGC TTG TAG TGC AT
	Nested 2	PfFD_F2	TGC GCA GCA AAA TTA GTC GAA
		PfFD_R2	CAT TCC CCA TTT CAA TCA TAT CCA
pfmdr2 (PF3D7_1447900)	Nested 1	Pfmdr2_F1	TTT GTG GCC AAG CAA AAG GA
		Pfmdr2_R1	TCT TTG TCG TTC TCC TCC TGA
	Nested 2	Pfmdr2_F8	AGA GGT ACC GAG AGT GCT AA
		Pfmdr2_R8	AGA GCA CAT GTT GTA CCT GGT T
pvcrt-o (PVX_087980)	Nested 1	Pvcrt_N2F	ACG GAA TCA ACC CGA ATC CA
		Pvcg10_R	AGT TTC CCT CTA CAC CCG
	Nested 2	Pvcrto_F	TCC TTG CCG CTG ATT CTA CG
		Pvcrto_R	GGT AAC GTT CAT CGG GGG TT
pvmdr1 (PVX_080100)	Nested 1	Pvmdr1_F3	GGA TAG TCA TGC CCC AGG ATT G
		Pvmdr1_R1	CTT ATA TAC GCC GTC CTG CAC
	Nested 2	Pvmdr1_F3	GGA TAG TCA TGC CCC AGG ATT G
		Pvmdr1_R3	CAT CAA CTT CCC GGC GTA GC
pvdhps (PVX_123230)	Nested 1	Pvdhps_F1	AGG AAG CCA TTC GCT CAA C
		Pvdhps_B	GAG ATT ACC CTA AGG TTG ATG TAT C
	Nested 2	Pvdhps_D	GGT TTA TTT GTC GAT CCT GTG
		Pvdhps_B	GAG ATT ACC CTA AGG TTG ATG TAT C
pvdhfr (PVX_089950)	Nested 1	Pvdhfr_F1	ATG GAG GAC CTT TCA GAT GTA TT
		Pvdhfr_N1R	CGG GTT TTT CTC CCC CAC TT
	Nested 2	Pvdhfr_F1	ATG GAG GAC CTT TCA GAT GTA TT
		Pvdhfr_R1	CCA CCT TGC TGT AAA CCA AAA AGT CCA GAG

Technical Appendix Table 2. Characteristics of 28 patients with asymptomatic malaria infection, Myanmar, 2015

Patient no.	ID	Village	Age, y	Sex	Occupation	PCR result	Microscopy result, parasites/µL
1	A954	Let Pa Dan	59	F	Dependent	*P. vivax*	NS*
2	A878	Let Pa Dan	24	F	Farmer	*P. vivax*	NS
3	A887	Let Pa Dan	23	F	Farmer	*P. vivax*	NS
4	A894	Let Pa Dan	52	M	Manual worker	*P. vivax*	NS
5	A903	Let Pa Dan	31	F	Farmer	*P. vivax*	NS
6	A905	Let Pa Dan	19	M	Student	*P. vivax*	NS
7	A910	Let Pa Dan	17	M	Manual worker	*P. vivax*	NS
8	A808	Maung Yone	43	M	Farmer	*P. falciparum*	NS
9	A799	Maung Yone	32	M	Manual worker	*P. malariae*	NS
10	A850	Maung Yone	56	M	Dependent	*P. malariae*	NS
11	A762	Maung Yone	28	F	Farmer	*P. vivax*	NS
12	A857	Maung Yone	40	M	Dependent	*P. vivax*	NS
13	A356	Sate Ka Lay	52	M	Manual worker	*P. falciparum*	NS
14	A349	Sate Ka Lay	56	F	Dependent	*P. vivax*	NS
15	A383	Sate Ka Lay	21	M	Student	*P. vivax*	NS
16	A353	Sate Ka Lay	25	M	Manual worker	*P. vivax*	580
17	A377	Sate Ka Lay	41	M	Manual worker	*P. vivax*	NS
18	A409	Tha-Yet-Chaung	35	M	Company staff	*P. vivax*	NS
19	A541	Tha-Yet-Chaung	26	F	Farmer	*P. falciparum*	NS
20	A548	Tha-Yet-Chaung	29	F	Farmer	*P. vivax*	NS
21	A624	Tha-Yet-Chaung	25	M	Farmer	*P. vivax*	1,200
22	A661	Tha-Yet-Chaung	29	M	Manual worker	*P. vivax*	NS
23	B1095	Wae Gyi	17	M	Taxi cycle driver	*P. falciparum*	NS
24	A968	Wae Gyi	18	M	Manual worker	*P. vivax*	NS
25	A031	Win Ka Nane	26	F	Farmer	*P. vivax*	NS
26	A035	Win Ka Nane	9	M	Student	*P. vivax*	NS
27	A107	Win Ka Nane	45	M	Manual worker	*P. vivax*	NS
28	A032	Win Ka Nane	30	M	Manual worker	*P. vivax*	NS

*NS, not seen by microscopy.

Technical Appendix Table 3. Mutations in drug resistance molecular markers for 4 patients with asymptomatic *P. falciparum* malaria, Myanmar, 2015

Patient no.	ID	K13 (kelch gene)*	*pfarps10*	*pffd*	*pfmdr2*
1	A356	P574**L**	V127**M**	D193**Y**	T484**I**
2	A541	C580**Y**	V127**M**	D193**Y**	T484**I**
3	A808	Wild	Wild	D193**Y**	T484**I**
4	B1095	Wild	Wild	Wild	T484**I**

*Mutant amino acids are shown in bold. All of the sequences are aligned with 3D7 sequences retrieved from plasmodb.org.
